# Development and Validation of an Automated High-Throughput System for Zebrafish *In Vivo* Screenings

**DOI:** 10.1371/journal.pone.0036690

**Published:** 2012-05-15

**Authors:** Ainhoa Letamendia, Celia Quevedo, Izaskun Ibarbia, Juan M. Virto, Olaia Holgado, Maria Diez, Juan Carlos Izpisua Belmonte, Carles Callol-Massot

**Affiliations:** 1 Biobide S.L., San Sebastian, Guipuzcoa, Spain; 2 Gene Expression Laboratory, The Salk Institute for Biological Studies, La Jolla, California, United States of America; 3 Center of Regenerative Medicine in Barcelona, Barcelona, Spain; University of Cincinnati, United States of America

## Abstract

The zebrafish is a vertebrate model compatible with the paradigms of drug discovery. The small size and transparency of zebrafish embryos make them amenable for the automation necessary in high-throughput screenings. We have developed an automated high-throughput platform for *in vivo* chemical screenings on zebrafish embryos that includes automated methods for embryo dispensation, compound delivery, incubation, imaging and analysis of the results. At present, two different assays to detect cardiotoxic compounds and angiogenesis inhibitors can be automatically run in the platform, showing the versatility of the system. A validation of these two assays with known positive and negative compounds, as well as a screening for the detection of unknown anti-angiogenic compounds, have been successfully carried out in the system developed. We present a totally automated platform that allows for high-throughput screenings in a vertebrate organism.

## Introduction

The development of small-molecule therapeutics by target-directed strategies has been accelerated thanks to advances in genomic research, combinatorial chemistry and laboratory automation with development of high and ultrahigh-throughput screening (HTS) technologies [1]. However, although the target based approach is clearly advantageous from a scientific and practical point of view, it does not translate into a high success rate for novel targets (only 3% of drugs designed against a novel target reach preclinical development) [2]. Because of these problems, the pharmaceutical industry is increasingly focusing on known targets and looking for new indications for existing drugs, but this can only be a temporary solution because the number of such possibilities is limited. The important question is whether other approaches might be more suitable for the identification of novel treatments. It has been proposed that better models for drug testing are needed to improve the discovery of new drug candidates and whole organisms could provide such models [3], [4]; [5].

An important advantage of organism-based chemical screening is the fact that compounds are tested in the context of an intact organism rather than under artificial *in vitro* conditions. The cells of a whole organism are in their normal context and are non-transformed. The embryo contains many distinct cell types, making it theoretically possible to identify compounds that have tissue-specific effects or demonstrate deleterious side effects on complex developmental or physiological processes. The presence of a whole organism’s metabolism permits the identification of compounds that become active as metabolites and the lead identification can be done without a detailed knowledge of the targets or molecular pathways implicated in a specific disease [6], [7]. Small molecules discovered by virtue of their ability to induce a specific desirable phenotype in a whole organism are likely to fulfill requirements needed by promising drug candidates to enter in clinical development. They have to be cell-permeable, devoid of obvious toxicities, effective and possess favorable pharmacodynamic and pharmacokinetic profiles. Drug discovery in the whole organism, therefore, combines screening and animal testing in one step [8]. Animal models to be employed for organism-based chemical screens have to be small, low cost and compatible with simple culture conditions.

The nematode *Caenorhabditis elegans* and the fruit fly *Drosophila melanogaster* are popular genetic model organisms that have been used as screening tools in drug discovery [9]. An important limitation of invertebrate models is, however, the fact that they lack many complex organs, such as a cardiovascular system, an immune system and kidneys, that are relevant to human biology and physiology [8]. Among the vertebrate models, zebrafish embryos are particularly well suited for whole organism chemical screening because they are small, can be obtained in high numbers, develop quickly (most organs are formed within 24 h) and are optically transparent [10]. Embryos fit readily into microwell plates and compounds can be directly applied into the embryo medium [11]. Some manual screenings have been reported using zebrafish embryos [12], [13], [6], [14], [15], [16]. Recently, investigators have begun to adapt zebrafish to HTS protocols [17], [18], [19], [20], [21]. In all these examples automated software able to analyze images (for compounds that alter angiogenesis [18], [19] or mutants with retinal axon guidance alterations [20] or software able to analyze heart rate [17] and locomotor activity [21]) have been developed. Nonetheless, automation and integration of the whole screening process, from the embryo dispensation, compound treatment, incubation, phenotypic readout and analyses of the results have never been reported for zebrafish.

The goal of this work was to develop a fully automated platform for *in vivo* high-content and high-throughput screenings using zebrafish embryos as a whole-organism model. We have focused on the development of two different bioassays based on their interest for pharmaceutical companies, the availability of zebrafish tools and the previous knowledge of the transgenic lines chosen.

First, a cardiotoxicity assay to detect compounds that inhibit the ether-a-go-go-related-gene (ERG) potassium channel was set up. ERG blockade leads to an impairment of potassium outflow from the cell and consequently a disruption of the action potential, which is related to a prolongation of the QT interval in the electrocardiogram. The QT prolongation detection has extensively been used as a cardiotoxicity marker [22]. Current systems for detection of human ERG (hERG) inhibition are in vitro models which have disadvantages such as lack of evidence for the crosstalk of mechanisms. Zebrafish have been proposed as an experimental model to determine zebrafish ERG (zERG) inhibition [23] as the main mechanism of action. zERG has been identified and an 80% homology to the human protein has been postulated [24]. Here we will use a transgenic line expressing a GFP protein under the control of a myocyte specific promoter (Cmlc2) to analyze the effect on the heart function of a battery of compounds.

Second, an efficacy assay for detection of compounds that inhibits angiogenesis has been developed. Angiogenesis, the process of new blood vessel formation from pre-existing ones, plays a key role in various physiological and pathological conditions [25]. Many of the genes involved in the angiogenesis process in higher vertebrates (vascular endothelial cell growth factors (VEGF), fibroblast growth factors (FGF), ephrin receptors, angiopoietins, etc) are conserved and have the same function in zebrafish [26], [27], [28], [29]. Blood flow begins in the zebrafish embryo at ∼ 24 hours post-fertilization (hpf). Shortly after this, the angiogenic vessels that perfuse the trunk of the embryo (intersegmental vessels, ISVs) sprout from the vasculogenic vessels (dorsal aorta (DA) and posterior cardinal vein (PCV)). The existence of transgenic zebrafish that express green fluorescent protein GFP under the control of specific promoters of the vascular system (i.e. Flk1 and Fli1), allows direct observation of ISVs development and defects in this process.

In this report, we present how the automated platform has been established. To show the flexibility of the platform designed, the two different bioassays described for detection of cardiotoxicity or angiogenesis inhibition have been successfully set up and validated. Our results support zebrafish embryos as an *in vivo* bioassay tool that can contribute to several aspects of the drug development process, including drug target identification, lead compound discovery and detection of drug toxicity.

## Results

Our HTS platform was designed to avoid manual manipulation of zebrafish embryos and achieve fully automated screening assays aiming for the highest possible throughput and minimizing human errors. The platform could be divided into a physical robotic system and an extensive software layer spread in different levels (1) the operation of the stand-alone devices, (2) the control of the platform, (3) the analysis of the images and the results and (4) the Laboratory Information Management System (LIMS).

### Instrument Configuration

**Figure 1:**
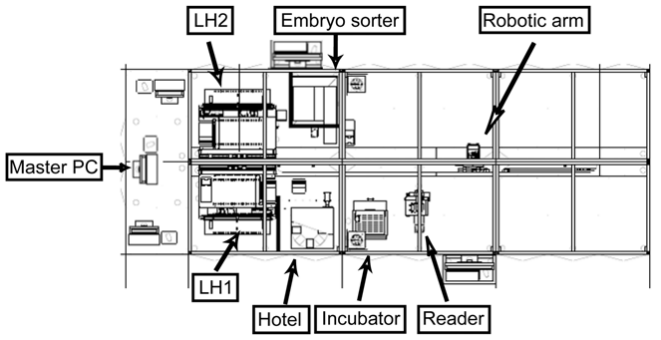
HTS platform layout. The figure shows all the components of the platform: master PC, embryo sorter, two liquid handlings (LH1 and LH2), plate hotel, incubator and reader. The robotic arm in the central part transports the plate to all the devices.

The robotic system is comprised of several devices (plate hotel, embryo sorter, liquid handling, automatic incubator and microscope) that were individually tested and optimized before final integration into the complete process. A robotic arm allows the automatic transport of the assay plates between instruments (Figure 1).

**Figure 2:**
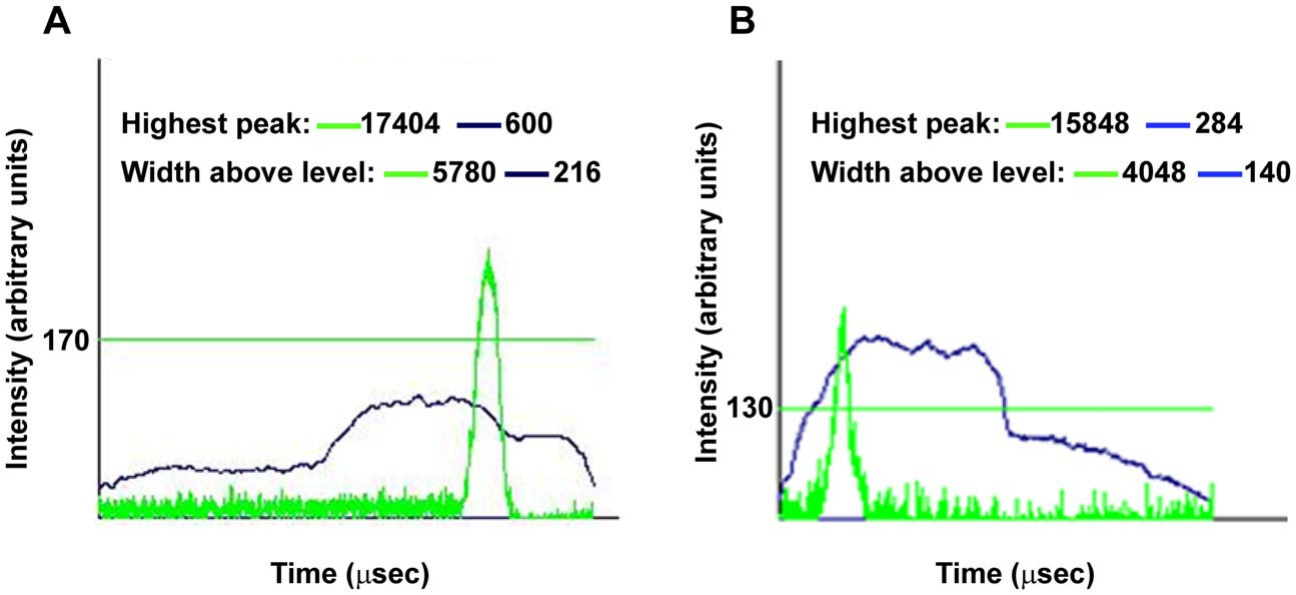
Sorter embryo profiles. The figure shows a representative example of one embryo profile from Cmlc2:GFP at hpf 48 hpf (A) or Flk1:GFP at 24 hpf (B), provided by COPAS sorter. Two parameters are represented in the Y axis, optical density (blue line) and intensity of the fluorescence (green line) that are scaled differently (maximum of 1.024 and 65.536 respectively). The particle size is measured as the time of detection and is represented in X axis. Sorted embryos should be in a defined interval of length and the fluorescence peak above the established threshold (green horizontal line) to be dispensed; (5000–7500 µs and 170 a.u for Cmlc2:GFP embryos and 3050–7000 µs and 130 a.u for Flk1:GFP embryos). a.u: arbitrary units.

The transgenic lines employed to automate the experiments expressed the fluorescent protein copGFP. To be able to dispense one embryo per well we optimized the different parameters affecting the sorter performance, which are: delay, sorting criteria and width. Optimizing the delay minimizes undesired results such as empty wells, doublets or triplets. The sorting criteria parameters chosen were the height of the GFP peak (indicates GFP intensity expression) and the particle size (indicates the length of the embryo) measured in the signal detected using the Profiler module (Figure 2 A, B). Proper thresholds were established to assure that only embryos in the correct stage showing enough fluorescence were dispensed. These values resulted in one third of the embryos located in the sorter reservoir (approximately 270 embryos) to be finally dispensed in the assay plate within 3 to 5 minutes with a rate of doublets/empties lower than 5% on average. After sorting, we evaluated heartbeat, morphology, embryo development and pigmentation and no differences were found compared to manual dispensation (data not shown).

Variation in volume of the dispensed drop had to be considered to establish the total volume in the well. The drop size, defined by the width parameter was monitored during the parameterization process. We determined that the variation of the drop size should not exceed 10% of the total final volume in the well. Since this variation was slightly higher in 48 hpf Cmlc2:copGFP embryos (73±12 µl for a final volume of 100 µl) a leveling step (described next) was introduced. In the case of 24 hpf Flk1:copGFP embryos, the variability of the drop obtained (40±7 µl in a final volume of 180 µl) satisfied the established criteria and no leveling step was necessary.

Two liquid handling devices were integrated in the platform to allow the performance of parallel operations, thus improving the throughput of the process. These two devices were used for the addition of compounds, the leveling step in the cardiotoxicity assay (aspiration of the exceeding volume dispensed with the embryo) and the tricaine addition in the angiogenesis assay.

A critical step during the platform integration was the image acquisition. The microscope and the camera were configured to scan the assay plate well by well. A macro for the scanning process and image acquisition of each assay type was developed using AxioVision software. For the cardiotoxicity assay, a video of the fluorescent heart was required for the posterior automated image analysis. First, the heart of the embryos had to be identified. For this purpose the 1.25× objective and the fluorescent light were used and the heart was identified by its intensity and area. Afterwards, the 10× objective was used to (1) check if the heart was still in the field of view (by measuring intensity and area) and (2) the autofocus was applied to get an in focus heart. The image containing the heart was cropped to avoid unnecessary information and a sequence of 512 frames at 30 frames per second was recorded.

The angiogenesis assay required a fluorescent image, then a scan per well was performed to identify the embryo. Once identified, the embryo was segmented according to determined thresholds of intensity and area, centered in the field of view and autofocused. The tail region of the embryo was focused avoiding the head area and a fluorescent image was acquired for processing by the image analysis software.

Finally, in both assays, cardiotoxicity and angiogenesis, a bright-field image of each well was taken to evaluate the embryos’ general condition.

### Software Development

In this section the overall control of the platform, the specific image analysis software developed for each assay and the data management are described.

The integration of all the devices required a control system programmed to automatically run the different assay types. It operates using a configuration file that specifies the features of an assay plate. In order to plan the process for a set of plates for an assay, a scheduler module was implemented. Each location corresponds to a task of the assay and is shown as a differently colored bar in the screen (Figure S1).

The image analysis program for the Cardiotoxicity assay was based on the processing of the videos to obtain a periodic signal of the cardiac motion automatically and independent to the embryo position. A custom-made analysis software (Cardio v3.0.0.5) was generated (in collaboration with the technological center Vicomtech) to detect the heart rate, the presence of alterations in the frequency of atrial and ventricular contraction (arrhythmia) and the absence of heartbeat (cardiac arrest).

**Figure 3:**
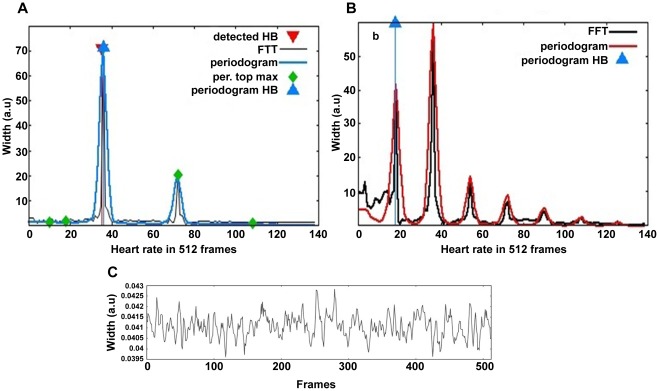
Image analysis of the heart rate. The figure shows the analysis of the heart rate of a representative control (A), arrhythmic heart (B) and cardiac arrest (C). OF and ED algorithms are applied for the analysis of each video. Only when the OF is above a specific threshold FFT is applied to ED. Control and arrhythmic hearts are classified based on the number of the significant frequencies detected in the FFT, one for control (A) two or more for arrhythmic (B). (C) OF representative of a dead embryo. In FFTs, Periodogram is shown in blue, Original Fourier Transform of the ED signal in black and Heartbeat is shown in red.

The detection of the heart beat frequency was based on the frequency spectrum analysis of the Euclidean Distance (ED) signal (built as the ED between the frames of the video referred to a reference frame calculated with the mean values of intensity for homogeneous zones). Obtaining the periodogram using the Discrete Fourier Transform (DFT), and modifying it to avoid undesired effects, the heart beat frequency was given by its first significant frequency (Figure 3 A).

For embryos with arrhythmia (A), the sinusoidal course of the ED signal was disrupted due to the irregular beating of the heart, i.e. the different beating frequency of each chamber. The classification of the arrhythmia cases was therefore based on the number of significant frequencies detected in the DFT of the ED signal (Figure 3 B).

For cardiac arrest (D) detection, a threshold was defined for the amplitude between maxima and minima of the Optical Flow signal (based in the calculation of a movement measure of brightness for each frame referred to a reference frame). The low level of movement that appeared in dead embryos produced low values of amplitude indicating cardiac arrest (Figure 3 C).

Reliability of the image analysis software was tested by comparing the software output to manual analysis of the videos. The data showed that the automatic calculation of the number of heartbeats coincided with manual counting in 98.4% of the cases. To measure the quality of detection of no effect, a total of 120 videos were analyzed and 93.3% of them were correctly classified. For arrhythmia and cardiac arrest effects, a total of 1324 and 1544 videos were evaluated and the percentages of those correctly classified were 95.6% and 97.5% respectively.

In the angiogenesis assay, the fluorescent images taken were processed using AxioVision 4.6.3 and a macro was developed to quantify an indirect measure of the area of the ISVs. Automatic image analysis is a fast method to identify positive compounds and can detect small variations in the width of the vessels indicating an effect that could not be detected by human eye. However, we usually include a manual counting to offer a more detailed measurement of effect.

**Figure 4:**
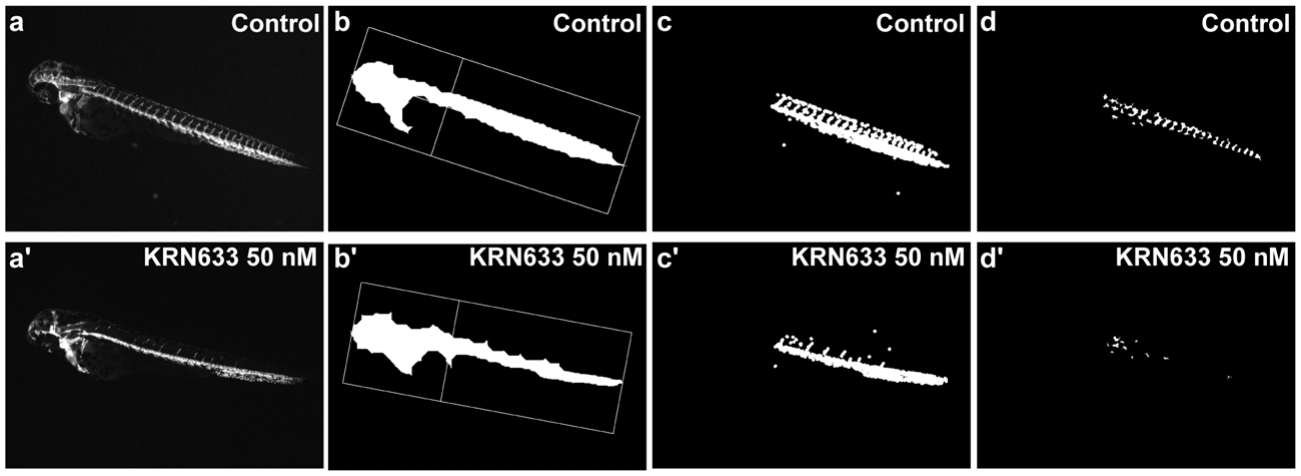
Images analysis for ISVs formation. The figure shows the different steps in the process of image analysis for the determination of the anti-angiogenesis effect. Control embryo (A, B, C and D); embryo treated with KRN633 (A’, B’, C’ and D’). (A) and (A’) are the original images acquired by the microscope; (B) and (B’) are images showing the segmentation between the head and the tail of the embryo to separate the ROI and measure the length; (C) and (C’) are images corresponding to an intermediate step in the filtering process where all the vessels in the tail can be identified; (D) and (D’) are images presenting the area enclosed between ISVs and DLAVs (in white) that are the ones to be quantified.

In the automatic analysis of angiogenesis, it is first necessary to perform a sequence of automated filtering functions on the image to extract information, define the region of interest (ROI), that in this case is the part of the tail containing the ISVs, and allow the measurement of the embryo length. Recovering a previous image, new filtering functions are applied to the ROI. A gradient image containing the ISVs, the DLAV (Dorsal Longitudinal Anastomotic Vessel), the PCV and the DA is created. Next, fluorescent areas that should not be quantified are discarded. Finally, only the areas comprised between the ISVs, the DLAV and the PCV or the DA are measured (Figure 4). In that way, when the vessels are incomplete or the DLAV is not present, an enclosed area is not present and therefore not measured. We have found that the inhibition of angiogenesis can be more efficiently detected in this way than when the direct measurement of the fluorescent area of the ISVs is applied.

The means obtained from the treated embryos were compared with the control mean. When statistically significant differences were detected a deeper analysis was done manually. In this case the parameters quantified were:

1. The total number of ISVs present in the trunk of the embryos (from where the largest bulge of the yolk ends to the end of the tail).
2. Number of complete ISVs (reached the dorsal part of the embryo and formed the DLAV).

The performance of the software developed for image analysis was checked over a training set of 450 tests (every test is a group of 10 embryos) manually evaluated that included control and treated embryos. Two different compounds (KRN633 and AG1478) at different concentrations were used to cover different degrees of effects. In 92% of the cases manual and automatic results coincided and, most importantly, only 1.8% of the tests in which an effect was induced, were not detected. In this case, a very slight anti-angiogenic action was always present.

In order to run the image analysis process off-line after the image acquisition and to generate the results file, a data processing program was developed. This program gets the data from the database and performs the statistical analysis (SPSS software). Finally, a result file (in Excel and Word formats) was released containing descriptive statistics and graphs.

Finally, a tailor made LIMS was developed (in collaboration with the Technological Center Tekniker) to support the activity of the laboratory and manage the information in the screening process. The LIMS receives data about compounds, embryos or plates (either manually or from other databases), tracks the information of the screening and shows all the relevant data of an assay and its final results files.

### Validation of the Cardiotoxicity Assay

The cardiotoxicity screening was performed at a specific and controlled temperature (27.5°C±0.5) since small changes in this parameter led to significant differences in heart rate (Figure S2, A). We also established that the interval for the stage of the embryos at the beginning of the analysis should be between 51 and 57 hpf since no differences in heart rate were found in embryos between these two stages (Figure S2, B).

Next, we selected three different effects that the system should identify (bradycardia, arrhythmia and cardiac arrest) in order to establish a useful assay for cardiotoxicity detection. To define a cutoff for bradycardia, the antipsychotic drug Thioridazine, which caused bradycardia in zebrafish embryos at 5 µM, was chosen. A group of approximately 100 embryos was treated with this compound. The receiver operating characteristic (ROC) curve exclusion model showed that 36.5 beat per 15 second was the threshold for bradycardia identification with sensitivity of 78% and a specificity of 70% (Figure S3). It is important to note that this cutoff point coincides when using other compounds such as Estradiol and Testosterone to induce bradycardia [30], [31] (data not shown).

Abnormal QT interval prolongation is a relevant cardiotoxicity marker because of its association with ventricular electric instability, which could induce polymorphic ventricular arrhythmias known as “torsade de pointes” [22]. Most QT-prolongation inducers exert their effect by inhibiting hERG, a delayed rectifier potassium channel. In zebrafish, inhibition of zERG using morpholinos leads to a specific phenotype called 2∶1 arrhythmia [24] that is the equivalent to an AV (Atrio-Ventricular) block in mammals. There is no evidence of a similar phenotype in other experimental models or when inhibiting other cardiac ion channels. This supports the linkage between type 2∶1 arrhythmia and zERG inhibition in zebrafish.

In order to detect zERG inhibition automatically, we analyzed 4 QT prolongation inducers (Terfenadine, Cisapride, Astemizole and Haloperidol) in a range of concentrations (0.1, 1, 5, 10, 20 and 50 µM) and compared them with 2 non-QT inducers (Digitoxine and Nitrendipine). Results showed that Terfenadine, Cisapride and Haloperidol were detected as potential QT-prolongation inducers at 5 µM and Haloperidol at 1 µM (Table 1). It is important to note that the mentioned concentrations do not reflect the real dose in the embryos. Bioavailability studies should be performed to evaluate it. In addition, a specific repertoire of phenotypes from no effect (Movie S1) and bradycardia (Movie S2) to heart failure (Movie S5) through severe atrio-ventricular 2∶1 arrhythmia (Movie S3) and ventricular failure (Movie S4) were observed, as previously reported [24]. This progression was not seen with other cardiotoxic drugs such as Digitoxine and Nitrendipine which do not cause a prolongation of the QT interval. This finding reveals that our method can specifically predict drug-induced QT prolongation mediated by a blockade of the HERG channel.

**Table 1:** Dose-effect analysis of QT and non-QT prolongation inducers.

COMPOUND	CONCENTRATION					
	0.1 µM	1 µM	5 µM	10 µM	20 µM	50 µM
Terfenadine	N	B	A	A	HF	HF
Cisapride	N	N	A	VF	HF	HF
Astemizole	N	A	HF	HF	HF	HF
Haloperidiol	N	N	A	VF	VF	HF
Digitoxin	ND	ND	N	B	HF	HF
Nitrendipine	ND	N	B	B	VF	HF

Four QT prolongation inducers (Terfenadine, Cisapride, Astemizole and Haloperidiol) and 2 non-QT prolongation inducers (Digitoxin and Nitrendipine) were tested on 48 hpf zebrafish embryos. Predominant observed effects are indicated: normal heart rate (N), bradycardia (B), 2∶1 arrhythmia (A), ventricle failure (VF), heart failure (HF).

To characterize the specificity and sensitivity of the method, 35 compounds were chosen according to their known therapeutic effects in humans and to the drug targets, including different ion channel proteins and regulatory pathways. The drugs were diluted in DMSO and blind screened in a concentration range between 1 and 200 µM. Based on their predominant effects they were automatically classified in 4 categories: No effect, cardiac arrest, bradycardia, and arrhythmia. The 35 compounds included: 17 QT prolongation related arrhythmia inducers (15 HERG channel blockers and 2 IKs channel blockers), 11 bradycardia inducers, 2 non-QT related arrhythmia inducers, 1 cardiac arrest inducer and 4 without effect. Fourteen out of 17 QT prolongation inducers were correctly detected, with them all being HERG channel inhibitors. All the other compounds were properly classified (Table 2). From these results we concluded that this method allows detecting of QT prolongation inducers mediated by an HERG blockade (observed as type 2∶1 arrhythmias) but also compounds that affect calcium channels (detected as bradycardia inducers) and sodium channels (detected as bradycardia in the case of the sodium channel blocker Lidocaine or as 2∶1 arrhythmia in the case of the sodium channel activator SDZ-201106). Moreover, the data suggest that it is possible to distinguish between QT related and non-QT related arrhythmias. The method showed a specificity of 90.3% and sensitivity of 100% for the compounds tested (Table 2).

**Table 2:** Screening of 34 small bioactive compounds.

REFERENCE COMPOUNDS	FAMILY	CARDIAC EFFECT IN HUMANS	CARDIOTOXIC EFFECT IN ZEBRAFISH	CLASSIFICATION
Lidocaine	Antiarrhythmic	Bradycardia	Bradycardia	TP
Flecainide	Antiarrhythmic	QT-prolong.	Arrhythmia 2∶1	TP
Propafenone	Antiarrhythmic	QT-prolong.	Arrhythmia 2∶1	TP
Amiodarone	Antiarrhythmic	QT-prolong.	Arrhythmia 2∶1	TP
Propranolol	Beta-Blocker	Bradycardia	Bradycardia	TP
Timolol	Beta-Blocker	Bradycardia	Bradycardia	TP
Thioridazine	Antipsychotic	QT-prolong.	Arrhythmia 2∶1	TP
Haloperidol	Antipsychotic	QT-prolong.	Arrhythmia 2∶1	TP
Ziprasidone	Antipsychotic	QT-prolong.	Bradycardia	FN
Pimozide	Antipsychotic	QT-prolong.	Arrhythmia 2∶1	TP
Sertindol	Antipsychotic	QT-prolong.	Arrhythmia 2∶1	TP
Risperidone	Antipsychotic	QT-prolong.	Arrhythmia 2∶1	TP
Fluoxetine	Antidepressant	Arrhythmia	Arrhythmia	TP
Doxorrubicin	Antitumoral	No effect	No effect	TN
Tamoxifen	Antitumoral	Arrhythmia	Arrhythmia	TP
Lapatinib	Antitumoral	No effect	No effect	TN
Verapamil	Calcium blocker	Bradycardia	Bradycardia	TP
Diltiazem	Calcium blocker	Bradycardia	Bradycardia	TP
Nitrendipine	Calcium blocker	Cardiac arrest	Cardiac arrest	TP
Terodiline	Calcium blocker	QT-prolong.	Arrhythmia 2∶1	TP
Pilocarpine	Colinergic agonist	No effect	No effect	TN
Nicotine	Colinergic agonist	Bradycardia	Bradycardia	TP
Astemizol	Antihistamine	QT-prolong.	Arrhythmia 2∶1	TP
Terfenadine	Antihistamine	QT-prolong.	Arrhythmia 2∶1	TP
Fexofenadine	Antihistamine	No effect	No effect	TN
Halofantrine	Antiinfective	QT-prolong.	Arrhythmia 2∶1	TP
Foscarnet	Antiinfective	Bradycardia	Bradycardia	TP
Cisapride	Gastrointestinal agent	QT-prolong.	Arrhythmia 2∶1	TP
Estradiol	Hormones	Bradycardia	Bradycardia	TP
Testosterone	Hormones	Bradycardia	Bradycardia	TP
L-768763	Iks blocker	QT-prolong.	Bradycardia	FN
Chromanol 293b	Iks blocker	QT-prolong.	No effect	FN
SDZ-201106	Ina opener	QT-prolong.	Arrhythmia 2∶1	TP
Digitoxin	Other	Bradycardia	Cardiac arrest	TP
Ketanserin	Other	Bradycardia	Bradycardia	TP

The table shows the compounds used for validation, their family and their cardiotoxic effects assessed in humans and zebrafish.TN (true negative), TP (true positive), FN (false negative), FP (false positive).

Next, we assessed the repeatability of the method. For this, three compounds (Lidocaine, Thioridazine and Haloperidol) with known effects on heart rate at 5 and 50 µM were tested. A total of 10 plates each containing 20 embryos per condition and 20 controls were analyzed. An interassay coefficient of variation (CV  =  standard deviation x100/mean) was calculated for each compound. The average coefficient of variation (CV) value was 5.2% (Table 3), indicating that the assay developed is highly repeatable.

**Table 3:** Repeatability assessment.

PREDOMINANT EFFECT	REFERENCE COMPOUNDS	INTER-ASSAY CV
No effect	Lidocaine 5 µM	3.2%
Bradycardia	Lidocaine 50 µM	2.5%
Bradycardia	Thioridazine 5 µM	3.7%
Arrhythmia	Thioridazine 50 µM	7.3%
Arrhythmia	Haloperidol 5 µM	5.4%
Cardiac arrest	Haloperidol 50 µM	9.3%

Calculation of the Coefficient of Variation (C.V) for ten independent experiments for the compounds and concentration indicated.

### Validation of the Angiogenesis Assay

To validate the angiogenesis assay developed in the HTS platform, 18 positive compounds were selected based on their capacity to inhibit known regulators of angiogenesis, including different tyrosine kinase inhibitors as VEGF receptors (VEGFRs), FGF receptors (FGFRs), PDGFR (platelet-derived growth factor receptor), etc. On the other hand, 10 compounds contained in the LOPAC^1280^ library previously tested in cellular HUVECs [32] and xenopus [33] screenings, which did not have an inhibitory action against angiogenesis, were used as negative items. The name and target of the compounds tested is shown in Table S1.

All these compounds were tested following the procedure described in the Methods section. A curve of six concentrations (0.01, 0.1, 1, 10, 30 and 100 µM) was performed for all of them. Only for the items that specifically inhibited angiogenesis, a second curve with specific concentrations was developed to calculate the IC50 for the two parameters manually quantified for the detection of the anti-angiogenic effect (total number and complete number of vessels). IC50 was calculated when a clear dose-dependent anti-angiogenesis effect was detected. Otherwise, the minimum dose at which the inhibition of angiogenesis was observed is shown (Table 4). 15 of the 18 positive compounds were detected as angiogenesis inhibitors in our assay (with only Tie-2 kinase inhibitor, Fumagillin and Hif-1 inhibitor not detected). None of the negative compounds tested showed any anti-angiogenesis effect. Therefore, the values of specificity and sensitivity are 83% and 100% respectively, what indicates that the assay developed allows for the detection of anti-angiogenic compounds with high specificity and sensibility in an *in vivo* model.

**Table 4:** Summary of the results obtained after angiogenesis assay validation.

COMPOUND	ANGIOG. INHIBITION	IC50 (µM)		EFECTIVE CONCENTRATION (µM)
		T.V.	C.V.	
**KRN633**	Yes	0.035	0.026	–
**ZD6474 (Vandetanib)**	Yes	–	–	100
**Sunitinib malate**	Yes	2.6	1.7	–
**Sorafenib Tosylate**	Yes	0.78	0.53	–
**PD173074**	Yes	–	–	100
**PD166866**	Yes	43.9	16	–
**AG-1296**	Yes	–	–	20
**PDGFR tyr kin inhibitor V**	Yes	0.19	0.14	–
**Tie2 Kinase inhibitor**	No	–	–	–
**Bosutinib**	Yes	–	–	50
**AG1478**	Yes	22.8	13	–
**Indirubin-3̀-oxime**	Yes	18.3	4.2	–
**Fumagillin**	No	–	–	–
**NS-398**	Yes	–	–	30
**HIF-1 Inhibitor**	No	–	–	–
**NVP-BEZ235**	Yes	–	–	10
**2-Methoxyestradiol**	Yes	30.6	10.2	–
**Paclitaxel**	Yes	–	–	?
**Tyrphostin AG490**	No	–	–	–
**Bestatin**	No	–	–	–
**Acetamide**	No	–	–	–
**E64**	No	–	–	–
**O6-benzylguanine**	No	–	–	–
**Cyclosporine A**	No	–	–	–
**4-Methylpyrazole hydrochloride**	No	–	–	–
**N-Acetyl-L-cysteine**	No	–	–	–
**Amiodarone hydrochloride**	No	–	–	–
**cis-Diammineplatinum(II) dichloride**	No	–	–	–

Table includes information about the presence or absence of an anti-angiogenic effect, the IC50 values when it was possible to calculate them or the minimum concentration that showed an effect when not. T.V: total number of vessels; C.V complete number of vessels.

To determine the assay robustness, 3 different experiments were carried out with some of the positive compounds representative of different targets. They were tested at one dose close to the IC50 or the effective concentration and the CV was calculated for the total fluorescence area of the ISVs and the total number of vessels quantified automatically and manually respectively. The results obtained (Table 5) indicate that the assay developed is highly repeatable with CV values always lower than 20%, except for KRN633 and Sorafenib, which were slightly higher when the fluorescence intensity was the parameter measured.

**Table 5:** Coefficient of Variation of the angiogenesis assay.

COMPOUND	TESTED CONCENTRATION (µM)	C.V. FLUORESCENT INTENSITY (%)	C.V. TOTAL VESSELS (%)
**KRN633**	**0.03**	**28.5**	**13.6**
**Sunitinib malate**	**2**	**7.6**	**2.2**
**PD166866**	**30**	**13.4**	**12.4**
**AG-1296**	**20**	**1.1**	**2.4**
**Bosutinib**	**60**	**7.6**	**0.7**
**AG1478**	**20**	**17.2**	**11.2**
**Sorafenib Tosylate**	**1**	**20.8**	**3.3**
**NS-398**	**40**	**11.6**	**3.2**
**2-Methoxyestradiol**	**10**	**13**	**2.6**

Calculation of the Coefficient of Variation of the indicated parameters calculated from data obtained after 3 independent experiments carried out with the positive compounds shown at one dose close to the IC50 or the effective concentration.

**Figure 5:**
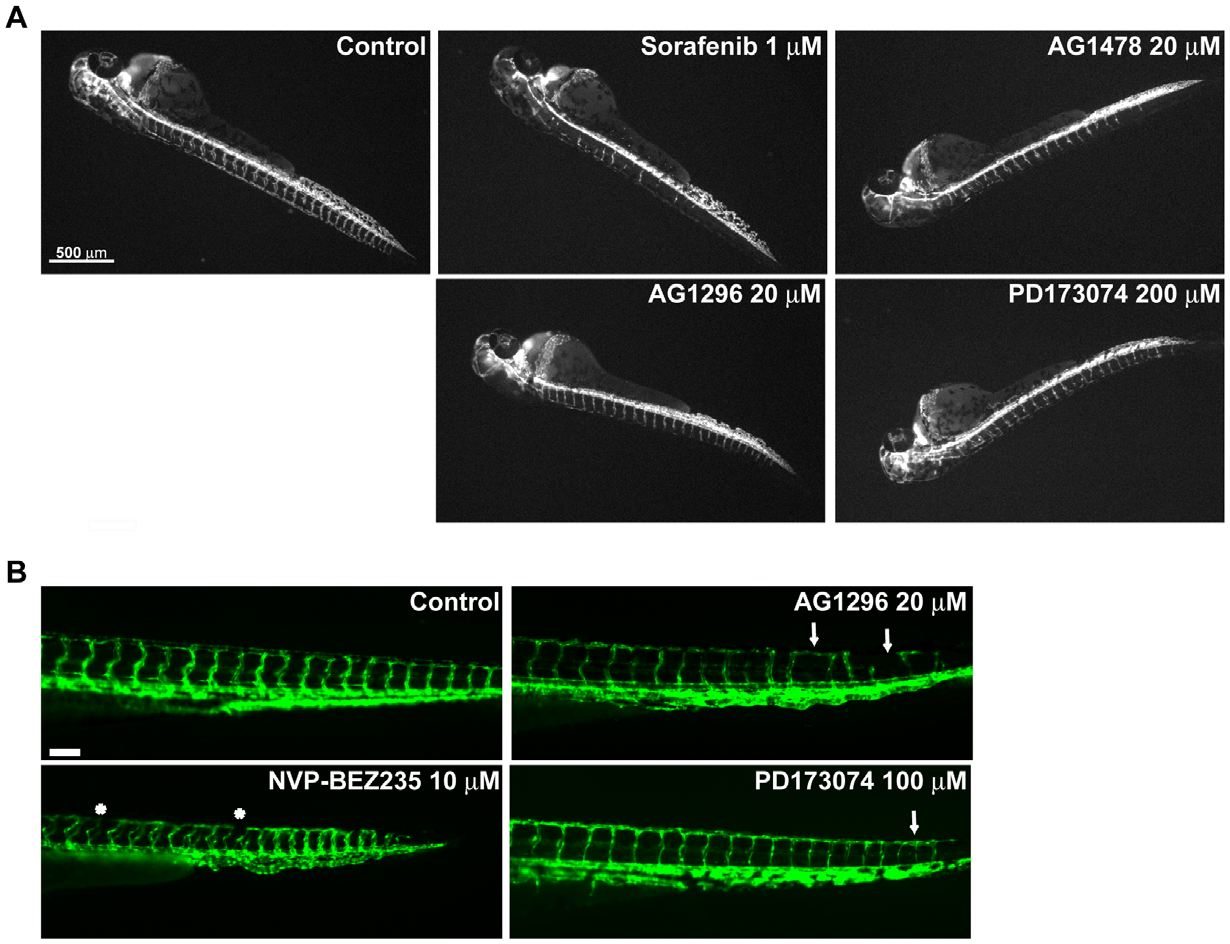
Two different phenotypes induced by angiogenesis inhibitors. 24 hpf zebrafish embryos were treated with different inhibitors and checked for angiogenesis defects at 48 hpf (A) or at 72 hpf (B) as described in material and methods section. (A) Representative fluorescent images of embryos treated with inhibitors that alter the development of ISVs at the level of sprouting and extension (superior panel) or that perturb later stages of maturation and/or DLAV formation (lower panel). Compared with control ones, treated embryos present a lower number of incomplete ISVs in the case of Sorafenib and AG1478 while AG1296 and PD173074 inhibit proper development of the DLAV and the ISVs looks thinner. (B) Representative fluorescent images of embryos treated with the indicated inhibitors showing the presence of thinner ISVs, some of them disrupted (arrows) and interrupted DLAV formation (asterisks).

Two clearly different phenotypes were detected for the positive anti-angiogenic compounds described before. In KRN633, ZD6474, Sunitinib, Sorafenib, PD166866, PDGFR tyrosine kinase inhibitor, AG1478, Indirrubin, 2-Methoxyestradiol and Paclitaxel treated embryos the formation of ISVs was perturbed with defective or total absence of sprouting. This effect was dose dependent and higher concentrations caused more intense phenotypes, with low numbers or absence of vessels and vessels stalled at the boundary between the notochord and the neural tube (Figure 5, A). However, compounds such as PD173074, AG-1296, NS-398, Bosutinib and NVP-BEZ235 showed a moderate effect that did not increase with higher doses, with little or no decrease in the total number of ISVs present but with the presence of thinner vessels (some of them incomplete) that do not always form the DLAV (Figure 5, A). While the first group of compounds is probably inhibiting initial essential steps in angiogenesis (sprouting and extension), the second group is probably more related to targets that participate in the proper maturation of the ISVs and/or DLAV formation.

The anti-angiogenic activity of the second group of compounds described was further demonstrated by studying their effects in embryos at 72 hpf, when circulation is already present. PD173074, AG-1296, Bosutinib and NVP-BEZ235 treated embryos showed impaired blood circulation and presented thinner ISVs that were disrupted in some cases (Figure 5 B). The absence of blood circulation indicates that even if vessels were initially developed they were not functional. NS-398 could not be tested at 72 hpf since it was lethal at 48 hpf doses, which inhibited angiogenesis. Therefore, different phenotypes associated with inhibition of different steps of angiogenesis regulation (sprouting and maturation) can be detected with this method.

Finally, a screening of 240 compounds representative of the BioFocus SoftFocus® library was carried out and the enzyme Phosphorilase Kinase was identified as a new potential targets for antitumoral therapeutics [34]. Therefore, the assay developed constitutes a powerful tool for library screening that will detect not only inhibitors for many of the known pathways that regulate angiogenesis, but also inhibitors of unknown regulators that could constitute new targets in drug development.

## Discussion

The results shown in this article support the use of zebrafish embryos in an HTS system. Zebrafish provide several advantages over other *in vivo* models. Their small size, transparency at early development stages, ability to grow in liquid medium and high number of offspring make them amenable to high-throughput work-flows. In addition, live animal screens seem to be ideal candidates for drug discovery strategies at the outset of the screening process as they are able to identify unwanted compounds at the earliest stages of drug development [35].

However, the lack of a totally automated system to distribute the embryos, add the treatments, acquire the images and analyze and store the data has prevented investigators from using zebrafish routinely in screening strategies due to the time-consuming features of these types of projects. Although other authors have focused on the image analysis for automation of zebrafish assays [36], [17], [18], [19], [20], [21], to our knowledge no data reporting a completely automated process has been published. Our goal in this work was to integrate all the operations required to complete an assay, providing the advantages of automated processes such as consistency between runs, automatic tracking of data, throughput and standardization, thus preventing variability due to human errors.

The design of the platform enables the use of the devices in several combinations and the addition of new instruments is possible. The equipment can be run integrated but also in stand-alone mode which is especially interesting in the case of the reader, image analysis and generation of the results for assays that require a repeatable readout but a complicated processing of the sample.

The developed system allows a throughput of 8 (cardiotoxicity) to 18 (angiogenesis) compounds per hour (with a sample size of 10 embryos) depending on the readout of the assay. The current design of the platform would duplicate the throughput of the assays by using a second microscope as the scanning of the plate is the bottleneck of the platform. Although automated systems *in vitro* provide higher throughputs, the amount of compounds analyzed is far higher than other in vivo assays in vertebrate models. High-throughput screens in cells to evaluate HERG inhibition have been developed [37], [38], [39]. These systems use hERG stably transfected cell lines and patch-clamp technique to evaluate current amplitude. In the method described by Guthrie et al., 37 compounds can be analyzed per hour. Each compound was used in 8 wells, generally allowing between 3 and 8 successful cells (data points) at the 1 concentration. Even if the throughputs of *in vitro* assays tend to be higher (the throughput of the patch clamp automated technique is 4 fold higher than our cardiotoxicity method) we have the advantages provided by an *in vivo* model such as testing compounds in an intact organism that allows to detection of tissue-specific effects that requires the interaction of different cell types as well as side effects during development. An example of the advantage of using zebrafish for cardiotoxicity detection was found in the classification of Verapamil. While *in vitro* assays target this compound as a potent HERG blocker [40], [41], studies in mammals show that it does not induce a prolongation of the QT-segment [42], [43]. This result agrees with our data indicating that Verapamil does not cause an AV block in zebrafish embryos. An explanation could be that although inhibition of HERG channels has been proposed as a primary test for screening purposes, several ion currents are involved in the generation of the cardiac potential. Then, the net effect caused by Verapamil leads to a shortening of the QT interval.

The preferred *in vitro* method for assessing angiogeneic regulators is endothelial cell tube formation assay [44], [45], [46], [47]. Although different endothelial cell tube formation assays that implicate one or more cellular types have been described as simple, rapid and quantitative, to our knowledge, the throughput obtained with these *in vitro* assays has never been published. Moreover, various days of culture are necessary to obtain a real tube formation, effects are usually difficult to quantify and not all of them are sufficiently robust for high-throughput screening. Apart from the general advantages of a whole organism assay mentioned above, the impairment of the proper development of ISVs in zebrafish embryos has been demonstrated as an excellent alternative for these *in vitro* assays [18], [19].

Next, both cardiotoxicity and angiogenesis assays were validated. For cardiotoxicity assessment, we defined the conditions to detect potential inducers of QT prolongation mediated by ERG blockade with a high specificity and sensitivity (14 out of 15 QT HERG mediated QT prolongation inducers were correctly detected). We also showed that compounds affecting calcium (Nitrendipine, Diltiazem, Verapamil) or inhibitors of sodium channels (Lidocaine) can be detected as bradycardia inducers. Moreover, the activation of sodium channels by treatment with the sodium channel opener SDZ-201106 caused type 2∶1 arrhythmias in zebrafish embryos and therefore could be classified as a potential QT-prolongation inducer. These data lead us to hypothesize that the characteristic type 2∶1 arrhythmias observed in zebrafish could be related to a delay in the action potential repolarization. Another possibility could be that SDZ-201106 might be blocking zERG channel as well as sodium channels. Further work should be done to test these hypotheses.

Among the 35 compounds analyzed, we have detected a low rate (8.5%) of false negatives (FN): Of the three FN found (Ziprasidone, L-768763 and Chromanol 293b), Ziprasidone was the only HERG blocker undetected out of 15 tested. L-768763 and Chromanol 293b are specific Iks channel blockers. The results with these drugs suggest that zebrafish embryos might not be expressing Iks or that this type of channel is not totally functional at the time of the assay (48 hpf). Again, new experiments should be conducted to demonstrate the functionality of Iks channels at different development stages of zebrafish embryos. All these data together indicates that our cardiotoxicity assay was successfully automated and that zebrafish embryos are an excellent model to detect cardiotoxic compounds acting through different targets.

The second assay developed was addressed to identify angiogenesis modulators. Angiogenesis is a cellular process, exquisitely regulated by which vessels already established, branch and invade tissues in response to normal or pathological stimulus [48]. The knowledge of the molecular mechanisms that regulate angiogenesis is clinically relevant and consequently, the detection of small molecules that inhibit angiogenesis is interesting for Pharmaceutical companies mainly focused in the development of products for oncology and ophthalmology.

As a result of the angiogenesis validation, 15 of the 18 positive drugs were detected as angiogenesis inhibitors. Moreover, two distinct phenotypes associated with the alteration of different steps during the angiogenesis process could also be differentiated.

All the compounds that inhibited VEGFRs (specifically or as one of the multiple tyrosine kinase targets) induced a clear perturbation in the sprouting of the endothelial cells with absent or uncompleted ISVs that were stalled half way. These results highlight the importance of VEGF growth factors and their receptors as the predominant regulators of vascular development [49], [50], also in zebrafish.

Two specific FGFRs (PD173074 for FGFR1 and 3 and PD166866 for FGFR1) and PDGFRs (AG1296 and PDGFR tyrosine kinase inhibitor V both for PDGFR α y β) inhibitors were also tested. While PD166866 and PDGFR tyrosine kinase inhibitor V induced a strong angiogenesis inhibition indicative of sprouting and extension defects, treatment with PD173074 and AG1296 resulted primarily in thinner ISVs rather than incomplete vessel formation. In fact, these vessels were not functional and started to be disrupted at 72 hpf. This discrepancy between phenotypes induced by different inhibitors for the same enzyme is not surprising. Kinases share similar structural frameworks in their active sites, so there is great potential for cross reactivity [51]. Our experience with other FGFR inhibitors tested (data not shown) and results obtained with a PDGFR inhibitor [52] or after silencing PDGFRβ2 with morpholino [53] indicates that probably PDGF and FGF regulate lumen formation in the process of maturation of the ISVs in zebrafish. In fact, different studies suggest that a combination of FGF2 and PDFG-BB synergistically promote formation of stable vessels in mammals [54], [55]. All these data suggest that the phenotype found with PD173074 and AG1296 is probably the one specific for the inhibition for PDGFR β2 and FGFR respectively.

Nonselective antiangiogenic agents that target Abl and Src tyr kinases, EGFR, cyclin-dependent kinases, cyclooxigenase-2 (COX-2), PI3K, and microtubules stabilization also exert an anti-angiogenic action in zebrafish embryos. However, inhibition of one of the main regulators of vascular development in mammals, Tie2 tyr kinase receptor, did not induce any angiogenesis defect after inhibition in our screening. This result agrees with previous reports in which a zebrafish mutant line for Tie2 kinase did not show any defect in ISVs formation [56] although it participates in heart development. Hif-1 inhibitor was also not detected in our validation. Although Hif-1 factor is essential for angiogenesis induction in response to hypoxia (condition found in solid tumors) [57] to our knowledge its role in developmental angiogenesis has never been described. Therefore, even if some pathways are not conserved between zebrafish and mammals or not all the regulators of pathologic or developmental angiogenesis are the same, the developed assay permits the detection of inhibitors of many of the main pathways that regulate angiogenesis.

Taken together, we have implemented an automated platform for running screenings using zebrafish embryos for which the most relevant advantages are the flexibility to implement different types of assays, the robustness of the methods developed and the scalability of the system. Importantly, the level of integration of both assays allows us to run the screenings almost unattended by laboratory technicians. Furthermore, we provided the results of the validations performed in two different assays, demonstrating that automation of screenings using a vertebrate model is feasible.

## Materials and Methods

### HTS Platform

The HTS platform integrates an embryo sorter adapted from the Copas XL from Union Biometrica with a larger embryo reservoir and specific sensors and alarms, two liquid handling devices (Starlet’s from Hamilton), a plate-feeder carrousel (Cytomat Hotel from Thermo), an automatic incubator (Cytomat from Thermo), a reader (fully motorized inverted microscope Axiovert 200 M from Zeiss and EMCCD camera from Hamamatsu) and a robotic arm (TX60 from Staubli) as shown in Figure 1. It also uses several computers that run the applications and are connected through the network. Finally, Biobide’s main servers hold the databases and LIMS (web based application). The Celerra NS20 system from EMC is used for storing the images and result files.

### Compounds Tested in Validation

*Angiogenesis validation:* NVP-BEZ235, Sorafenib p-Toluenesulfonate salt, Bosutinib and Sunitinib malate salt were purchased from LC Laboratories; HIF-1 Inhibitor, Paclitaxel, AG1478, PD166866, Tie2 Kinase Inhibitor, 2-Methoxyestradiol and KRN633 from Calbiochem; NS-398, AG-1296, PD173074 from Cayman Chemical; Fumagillin from Tocris bioscience; PDGFR Tyrosine Kinase Inhibitor V from Merck; ZD6474 from Selleck. The rest of the compounds were purchased from Sigma-Aldrich. *Cardiotoxicity validation:* All compounds were obtained from Sigma, except SDZ-201106 and Lapatinib that were obtained from Biomol and Selleck respectively. L-768763 was kindly gifted by Merck.

### Zebrafish Husbandry and Transgenic Lines

Adult zebrafish were housed and maintained in accordance with standard procedures. All experiments were performed in compliance with the local animal welfare regulations and have been approved by Biobide’s ethical committee. Embryos were collected and raised in E3 media according to standard protocol (Nusslein-Volhard, 2002). For the angiogenesis assay TG(*Flk1*:copGFP) and TG(*Fli1*:EGFP) (Lawson et al, 2002) transgenic zebrafish lines that express green fluorescent protein GFP under the control of specific promoters of the vascular system, were used. For the cardiotoxicity assay transgenic zebrafish embryos expressing CopGFP under the cardiac-specific promoter Cmlc2 were used.

### Cardiotoxicity Assay

Embryos at 48 hpf were automatically dispensed in a 96 well plate discarding exterior columns and rows and volume was leveled to 50µl with E3. Compounds were prepared 200 times concentrated in DMSO. A 1∶100 dilution was automatically made from the stock and 50 µl were added to each embryo to reach the final concentration. Then, embryos were incubated at 28.5°C for 3 hours. Embryos were not anesthetized to avoid potential beating alterations.The microscope scanned the wells by columns and a movie of the beating heart was recorded (512 frames) at 27.5°C±0.5 in all cases (except for comparison of temperature variable). Embryos that moved during video recording were discarded. *Analysable* videos (67%) were processed by using non-commercial Cardio v3.0.0.5 software. One video per embryo from a minimum of 10 embryos in each treated group were necessary for statistical analysis.

### Angiogenesis Assay

For the angiogenesis assay embryos were obtained and kept at 26.7°C until the time of treatment (22–26 hpf). At this time, chorions were removed with protease from Streptomyces griseus (Sigma-Aldrich) (1.5 mg/ml for 6 minutes) and the embryos were sorted and dispensed 1 per well in a 96 well assay plate. Each experimental condition was tested in a row with ten embryos. Embryos were dispensed in an average final volume of 90 µl of E3 media. 90 µl of the test items (DMSO and positive control KRN633 included) previously diluted 100 times were dispensed into each well of the assay plate containing the embryos to get the final volume of 180 µl and the desired final concentration.

In the case of Paclitaxel, because the compound precipitated after the dilution in E3 medium, and the maximum dose tested (10 µM) was not enough to induce inhibition of angiogenesis, an injection of 10 nl of the compound at 10 mM diluted in DMSO was directly injected in the yolk.

The assay plate was incubated at 28.5° during 23–26 hours. After that, embryos were anesthetized with 10 µl tricaine (final concentration 0.12%) to prevent their movement and after 12 minutes of incubation the assay plate was placed in the microscope (Axiovert 200 M). The microscope scanned the wells by columns and a bright field image and a fluorescent image (2.5×magnification) were acquired per well. Then, the image analysis was performed and the result file generated.

### Embryo Analysis at 72 hpf

Embryos from the Flk1:copGFP line were treated with the indicated compounds at 22–23 hpf in 500 µl of E3 in p24 well plates (5 embryos per well). After 48 hours of treatment embryos were anesthetized with 10 µl Tricaine (final concentration 0.02%) and analyzed for the presence of blood circulation and ISVs development using a Leica M205 FA stereoscope equipped with a Hamamatsu C10600 camera.

### Statistical Analysis

Statistical analysis and graphical representation of the data were performed using SSPS (Statistical Package for the Social Sciences) software and Prism (Graph Pad Software) for IC50 calculations (non linear regression, sigmoidal dose-response with variable slope). As heartbeat followed a non-Gaussian distribution (skewness value -0.9, p<0,001), a U-Mann-Whitney test was applied to compare treated versus control groups. In the angiogenesis assay, for comparisons between two groups, two-tailed *t*-test was used assuming unequal variances. For comparisons between more than two groups one-way analysis of variance followed by Dunnett’s post-test was applied to compare each data point with vehicle control.

## Supporting Information

Figure S1 **Scheduling of the assays developed.** The figure shows an example of scheduling of a typical cardiotoxicity (A) or angiogenesis assay (B). Each color corresponds to a different task: dispensation of the embryos (yellow), addition of compound (blue), drug incubation (green), read out (pink) and transport (grey). The microscope is the bottle-neck of the platform, thus each plate is planned to reach the microscope after the reading of the previous one.
(PPTX)

Figure S2 **Effect of the read out temperature and embryo stage on heart rate.** One cell stage embryos were incubated at 28.5°C until they reached 48hpf (A) and 48, 51 or 54 hpf (B), and plated as described in Material and Methods section. Heart rates were measured at 51 hpf at two different temperatures 24.5°C and 27.5°C (A) or only at 27.5°C (B). (A) A total of 4 plates were analyzed for each temperature. Each dot in the graph represents the heart rate of a single embryo. At 27.5°C the heart rate was significantly higher that at 24. 5°C (p<0.001). (B) A total of 4 plates were analyzed per stage. As shown in the graph no statistically significant differences were found between the three stages in the heartbeat.
(PPTX)

Figure S3 **Determination of bradycardia threshold.** 48 hpf embryos were treated as described in Material and Methods with 5 µM Thioridazine. The compound was tested on approximately 100 embryos distributed in 10 plates and the graph represents the ROC curve. 36.5 was chosen as the discriminatory value between control and bradycardic heart rates with sensitivity of 78% and specificity of 70%.
(PPTX)

Table S1 Compounds checked in system validation and their targets.
(PPTX)

Movie S1 Heart beating of an untreated embryo.
(AVI)

Movie S2 Heart beating of an embryo treated with 50 µM Lidocaine.
(AVI)

Movie S3 Heart beating of an embryo treated with 5 µM Terfenadine.
(AVI)

Movie S4 Heart beating of an embryo treated with 10 µM Cisapride.
(AVI)

Movie S5 Heart beating of an embryo treated with 50 µM Nitrendipine.
(AVI)
