# Supplementary figures and images for: Development and Validation of an Automated High-Throughput System for Zebrafish In Vivo Screenings
Source: PLoS One. 2012 May 15;7(5):e36690. doi: 10.1371/journal.pone.0036690 (PMC3352927; doi:10.1371/journal.pone.0036690)

## Slide 1
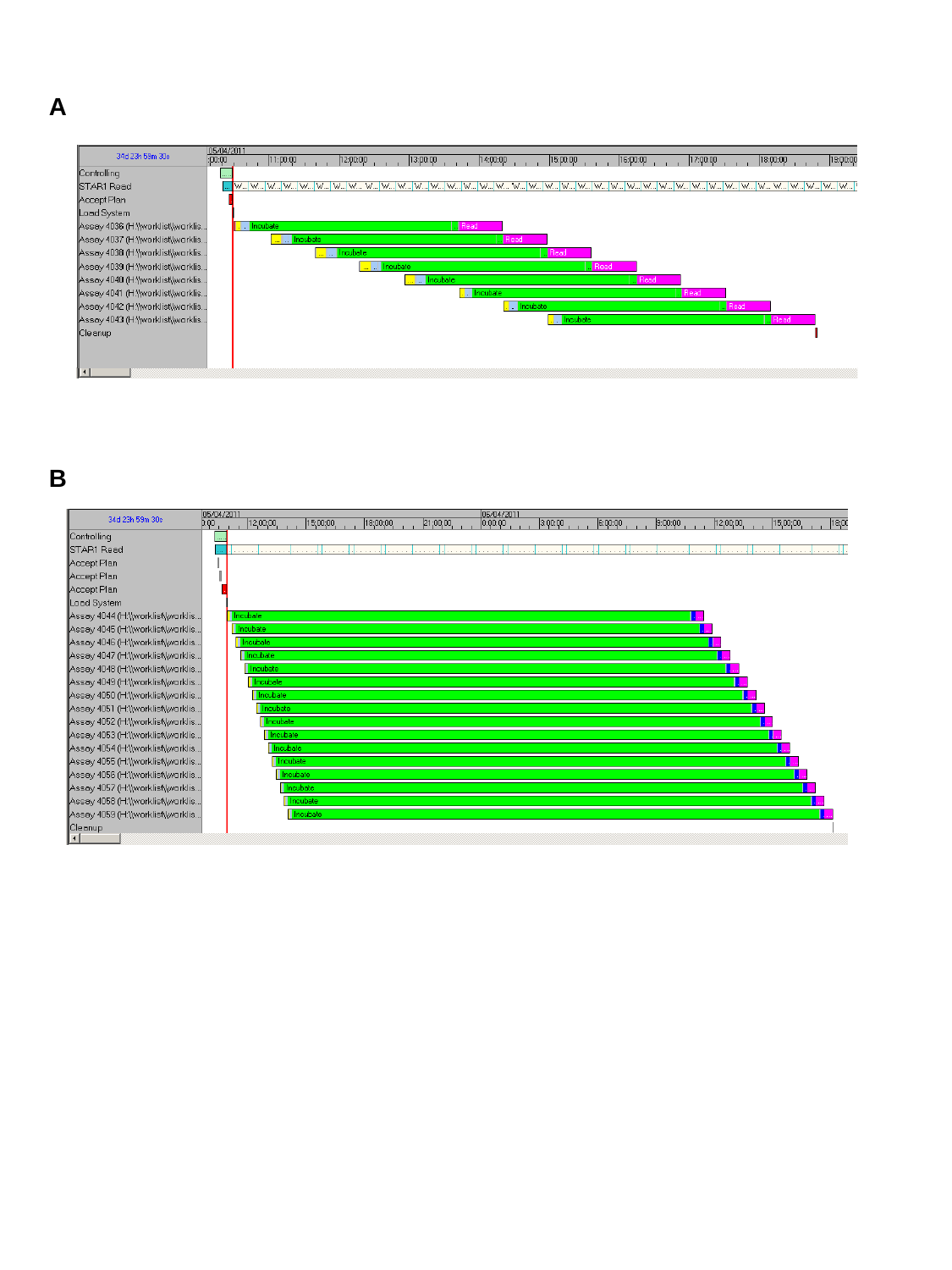

A
B

Supplement: Figure S1 — Scheduling of the assays developed. The figure shows an example of scheduling of a typical cardiotoxicity (A) or angiogenesis assay (B). Each color corresponds to a different task: dispensation of the embryos (yellow), addition of compound (blue), drug incubation (green), read out (pink) and transport (grey). The microscope is the bottle-neck of the platform, thus each plate is planned to reach the microscope after the reading of the previous one. (PPTX) [file pone.0036690.s001.pptx]

## Slide 1
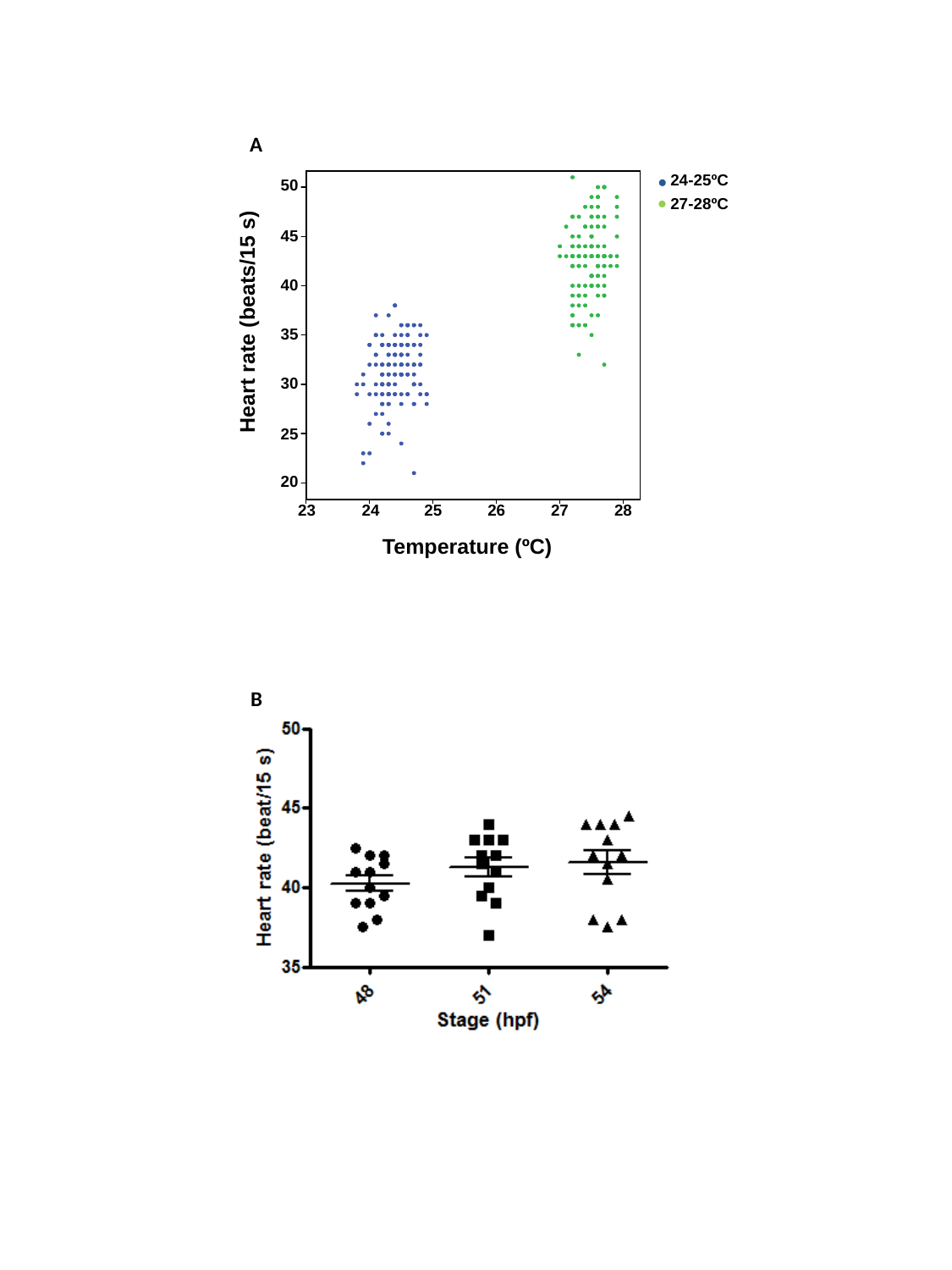

A
24-25ºC
50
27-28ºC
45
40
Heart rate (beats/15 s)
35
30
25
20
23
24
25
26
27
28
Temperature (ºC)
B

Supplement: Figure S2 — Effect of the read out temperature and embryo stage on heart rate. One cell stage embryos were incubated at 28.5°C until they reached 48hpf (A) and 48, 51 or 54 hpf (B), and plated as described in Material and Methods section. Heart rates were measured at 51 hpf at two different temperatures 24.5°C and 27.5°C (A) or only at 27.5°C (B). (A) A total of 4 plates were analyzed for each temperature. Each dot in the graph represents the heart rate of a single embryo. At 27.5°C the heart rate was significantly higher that at 24. 5°C (p<0.001). (B) A total of 4 plates were analyzed per stage. As shown in the graph no statistically significant differences were found between the three stages in the heartbeat. (PPTX) [file pone.0036690.s002.pptx]

## Slide 1
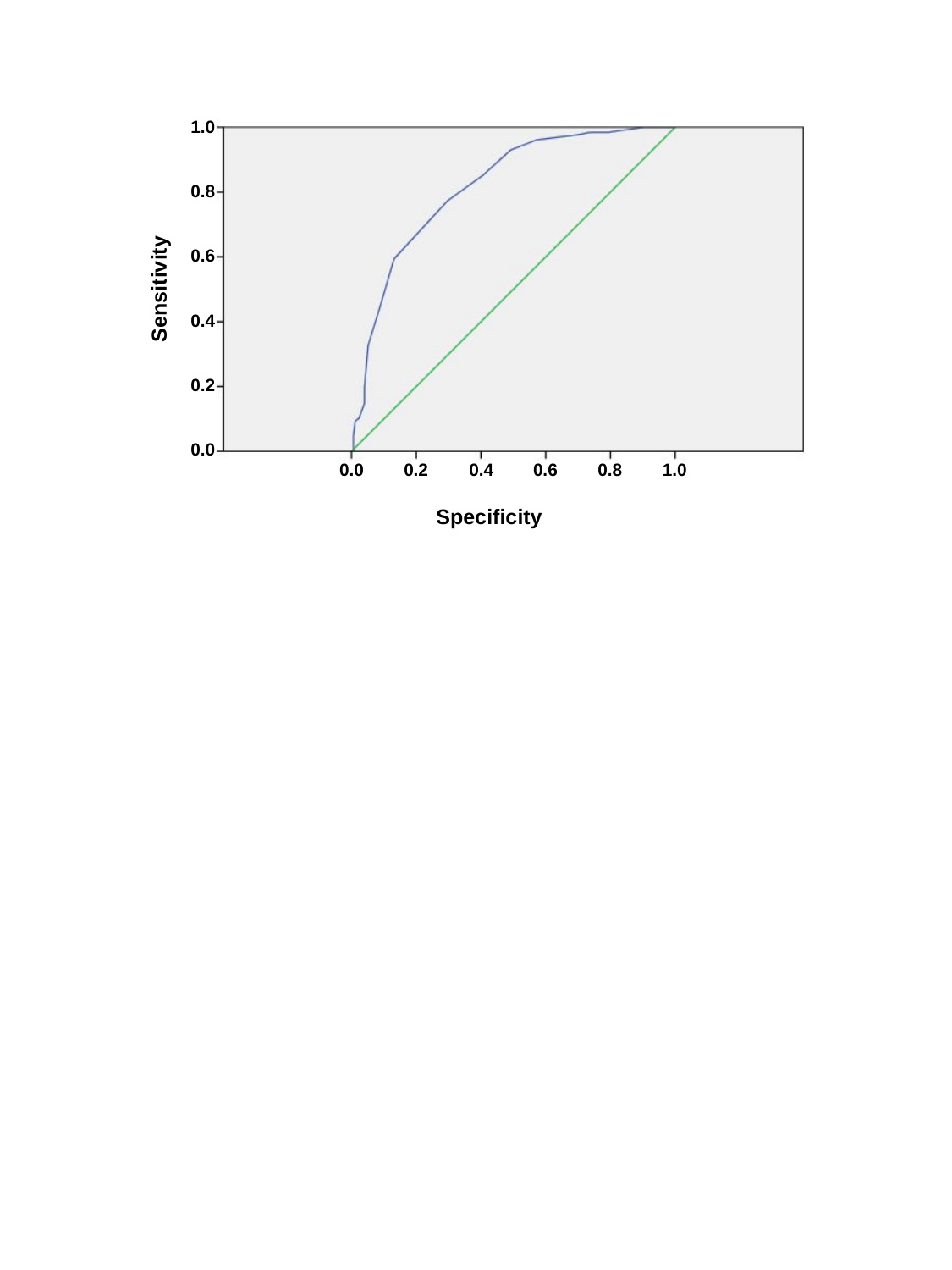

1.0
0.8
0.6
Sensitivity
0.4
0.2
0.0
0.0
0.2
0.4
0.6
0.8
1.0
Specificity

Supplement: Figure S3 — Determination of bradycardia threshold. 48 hpf embryos were treated as described in Material and Methods with 5 µM Thioridazine. The compound was tested on approximately 100 embryos distributed in 10 plates and the graph represents the ROC curve. 36.5 was chosen as the discriminatory value between control and bradycardic heart rates with sensitivity of 78% and specificity of 70%. (PPTX) [file pone.0036690.s003.pptx]

## Slide 1
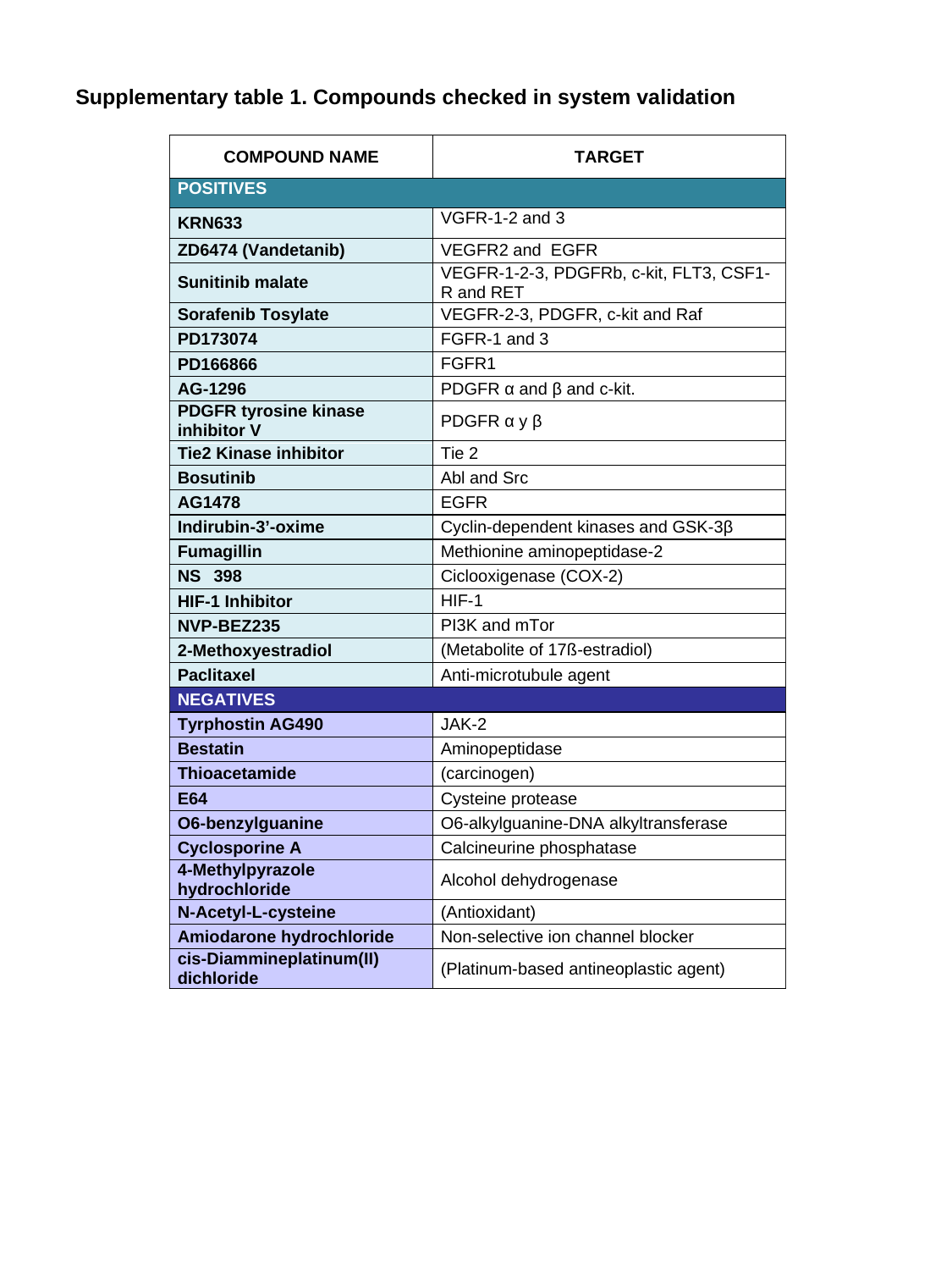

Supplementary table 1. Compounds checked in system validation

Supplement: Table S1 — Compounds checked in system validation and their targets. (PPTX) [file pone.0036690.s004.pptx]
